# Exercise-Induced Hypoalgesia Following Proprioceptive Neuromuscular Facilitation and Resistance Training Among Individuals With Shoulder Myofascial Pain: Randomized Controlled Trial

**DOI:** 10.2196/40747

**Published:** 2022-12-27

**Authors:** Zi-Han Xu, Nan An, Zi-Ru Wang

**Affiliations:** 1 School of Sport Medicine and Rehabilitation Beijing Sport University Beijing China

**Keywords:** exercise induced hypoalgesia, proprioceptive neuromuscular facilitation, PNF, resistance exercise, conditioned pain modulation, myofascial pain syndrome, resistance training, hypoalgesia, exercise-induced hypoalgesia, shoulder myofascial pain, myofascial pain, pain management, chronic pain, musculoskeletal pain, physical therapy, physiotherapy, shoulder pain, upper back pain, exercise, pain

## Abstract

**Background:**

Various exercises can attenuate pain perception in healthy individuals and may interact with the descending pain modulation in the central nervous system. However, the analgesic effects of exercise in patients with myofascial pain can be disrupted by the pathological changes during chronic pain conditions. Thus, the exercises targeted on the facilitation of the sensory-motor interaction may have a positive impact on the restoration of the descending pain modulation and the analgesia effects.

**Objective:**

This paper estimates the effect of proprioceptive neuromuscular facilitation (PNF) and resistance training on exercise-induced hypoalgesia (EIH) and conditioned pain modulation (CPM) among patients with myofascial pain syndrome.

**Methods:**

A total of 76 female patients with myofascial pain syndrome (aged 18-30 years), with the pain in the upper trapezius and a visual analog scale score of greater than 30/100 mm, were enrolled in the study. Participants were randomly assigned into 3 intervention groups, including isometric (n=18, 24%), isotonic (n=19, 25%), and PNF (n=20, 26%) exercises, as well as 1 control group (n=19, 25%) with no intervention. Pressure pain threshold and the CPM responses at the myofascial trigger point, arm, and leg sites were assessed before and after the exercise session. The effective EIH response was reflected in the improvement of pressure pain thresholds.

**Results:**

There was an increase in pressure pain thresholds and CPM responses at trigger point (*P*<.001 and *P*<.001), arm (*P*<.001 and *P*<.001), and leg sites (*P*<.001 and *P*=.03) in participants who performed PNF and isotonic exercise, while the isometric exercise only increased pressure pain thresholds at leg sites (*P*=.03). Compared with the control group, both the isotonic (*P*=.02) and PNF (*P*<.001) groups showed greater EIH responses at the trigger points. In comparison to the control group, only the PNF exercise (*P*=.01) significantly improved pressure pain thresholds and CPM responses at arm and leg sites compared to the control group.

**Conclusions:**

PNF, isotonic, and isometric exercises could lead to local and global EIH effects. The improvement in CPM response following PNF and isotonic exercises suggested that the EIH mechanisms of different resistance exercises may be attributed to the enhancement of the endogenous pain modulation via the motor-sensory interaction from the additional eccentric and dynamic muscle contraction.

**Trial Registration:**

Chinese Clinical Trial Registry ChiCtr202111090819166165; https://tinyurl.com/2ab93p7n

## Introduction

Exercise therapy has been recommended for the management of chronic pain [1] regarding its advantages in efficiency, cost, safety, and the exercise-induced hypoalgesia effect (EIH) [2] in both healthy people and individuals with pain. Both aerobic and resistance exercises may attenuate pain perception globally by enhancing endogenous analgesia [3] via descending pain modulation. However, patients with myofascial pain syndrome (MPS) always demonstrate dysfunction in descending pain modulation [4], which could stimulate and disrupt pain perception and even lead to the attenuation of the hypoalgesia effect induced by maximal and submaximal aerobic [5] or resistance exercises [6].

The altered endogenous pain modulation may be the reason for impaired EIH for patients with MPS, because they showed both local and global hyperalgesia. Normally, the sufficient noxious or nonnoxious input from the C afferent fibers can activate the thalamic mediodorsal nucleus and ventromedial nucleus [7] at different thresholds, triggering the descending facilitation (low thresholds) or inhibition (high thresholds) [8], respectively. Moreover, the ON or OFF cells in the periaqueductal gray [9], locus coeruleus, and rostral ventromedial medulla [10], which are projected to the spinal dorsal horn, can either activate the N-methyl-D-aspartate receptors [11] to facilitate the ascending nociceptive signals or release the endogenous opioid [12], 5-hydroxytryptamine (5-HT) [13], and noradrenaline [14] to inhibit the nociceptive signals. Meanwhile, the pain-related brain regions, including the primary motor cortex [15], anterior cingulate cortex [16], and insula cortex [17], can also participate in the descending modulation of pain through the motor-sensory interaction. However, the prolonged pain condition, especially MPS, can develop into central sensitization, where the balance between the descending facilitation and inhibition is disrupted and may damage the EIH effects.

With respect to the pathway of endogenous pain modulation, it is believed that the sensory input during the exercise, especially the proprioception and C fibers, can play a critical role in the EIH. Thus, proprioceptive neuromuscular facilitation (PNF) exercise [18], which can enhance the C fibers [19] and proprioception [20] inputs through the combination of various eccentric and dynamic exercises, may have additional positive effects on EIH in people with chronic pain; this is while the preliminary therapeutic effect of PNF on the pain of patients with MPS [21] has also been demonstrated. The type of muscle contraction can affect EIH in chronic pain. Specifically, isometric exercise could attenuate pain sensitivity in shoulder pain but not in fibromyalgia [22], while the isotonic exercise performed in nonpainful limbs could reduce pain perception in the case of chronic knee pain [23]. However, few studies have compared the effects of various resistance exercises on EIH in MPS. Moreover, the relationship between exercise type and endogenous analgesia features regarding EIH in MPS also needs further investigation.

In addition, conditioned pain modulation (CPM) [24] has been applied to evaluate the descending pain modulation in patients with chronic musculoskeletal pain. Additionally, the impairment of endogenous analgesia of MPS [25] was demonstrated by the attenuation of CPM responses. CPM has been shown to predict EIH in patients with knee osteoarthritis receiving bicycling and isometric exercise [26], while EIH has similar effects compared to CPM in healthy individuals [27]. However, the relationship between descending pain modulation and EIH has not yet been investigated in patients with MPS intervened by PNF and other resistance exercises.

Therefore, this pilot study aims to compare short-term EIH responses following PNF, isotonic, and isometric resistance exercises, as well as to investigate the relationship between EIH and descending pain modulation determined by CPM in patients with MPS. It was hypothesized that the PNF and all resistance exercises would have a higher EIH response compared with a blank control in individuals with MPS. The EIH and CPM response at baseline would be relatively lower in patients who have MPS with impaired descending pain modulation but would increase or be restored following PNF and resistance exercise intervention performed on the affected areas.

## Methods

### Ethics Approval

This study has been approved by the Sports Science Experimental Ethics Committee of Beijing Sport University (ethics approval number: 2021153H) and registered in Chinese Clinical Trial Registry (registration number: ChiCtr202111090819166165).

### Participants

A total of 76 female students (aged 18-30 years) from Beijing Sport University with shoulder MPS were enrolled in this study. This study selected female participants for the reason that EIH is potentially sex-related and is more consistently observed in women [28]. According to the pressure pain threshold (PPT) changes seen in previous studies [27,29], we used G-Power software with *F* test and ANCOVA parameters to calculate the sample size. The total samples of this study should be a minimum of 76 participants in the 3 groups, or 19 participants in each group.

The following inclusion criteria [30] were used to choose the participants: (1) reported shoulder pain persisting for at least 4 weeks up to 3 months and (2) had at least 1 latent trigger point on any side of the upper trapezius. The diagnosis of MPS adhered to the following standards: (1) palpation of a taut band, (2) identification of an exquisitely tender nodule (ie, the myofascial trigger points in the taut band), (3) reproduction of the patient’s symptomatic pain with sustained pressure, and (4) the local twitch response. The threshold value of the visual analog scale (VAS) for the MPS is set at 30 mm/100mm. If multiple trigger points were detected in a participant, the trigger point with the lowest threshold of the pressure pain would be selected.

Individuals were excluded if they met the following standards: (1) confirmed or suspected spinal or shoulder injury, dislocation, and fracture or inflammatory or infective diseases; (2) had a history of spinal or shoulder surgery within 12 months, or other physical treatment within 1 month; and (3) presentation of cardiovascular conditions, psychosis, depression, cognitive impairment, or taking drugs for antidepressant or anticonvulsant treatments, which would be carefully screened by a certified physician to ensure the safety of the intervention. The self-rating depression scale, self-rating anxiety scale, and brief psychiatric rating scale were applied and assessed by the physician during the screening periods.

All participants were randomly allocated into one of the four groups, as follows: group A (isometric exercise), group B (isotonic exercise), group C (PNF exercise), and group D (control). The randomized sequences were generated by a computer. All of the participants were labeled from number 01 to 76; then, the sequence was randomized using Excel software (Microsoft Corp) and allocated following the A-B-C-D circulation order. AN and XZH screened the participants.

### Procedures

This study is designed as a randomized controlled trial. Participants who were included in this study were invited to perform exercise interventions of either isometric (group A), isotonic (group B), or PNF (group C) exercises, while the participants in control (group D) would rest for 15 minutes during the intervention session. Each exercise consists of 2 scapula movements and 1 shoulder movement. The intensity of exercise was set as 60% maximum voluntary contraction (MVC) to avoid pain in the context of significant analgesic effects seen in previous studies on EIH [27] and PNF [31-33], and it could be adjusted to the subpain threshold if participants reported pain during the exercise.

The CPM and EIH responses (measured by PPT of trigger point and other remote limbs) were assessed before and after the exercise session as outcome measurements. The VAS, height, weight and duration of shoulder pain were also collected before the intervention. The VAS was measured using a scale printed with a line ranging from 0 mm (no pain) to 100 mm (worst pain), and participants were asked to locate a point on the line to rate their current pain level. The VAS would be only measured as the baseline characteristics and would not be considered as an outcome measurement.

**Figure 1:**
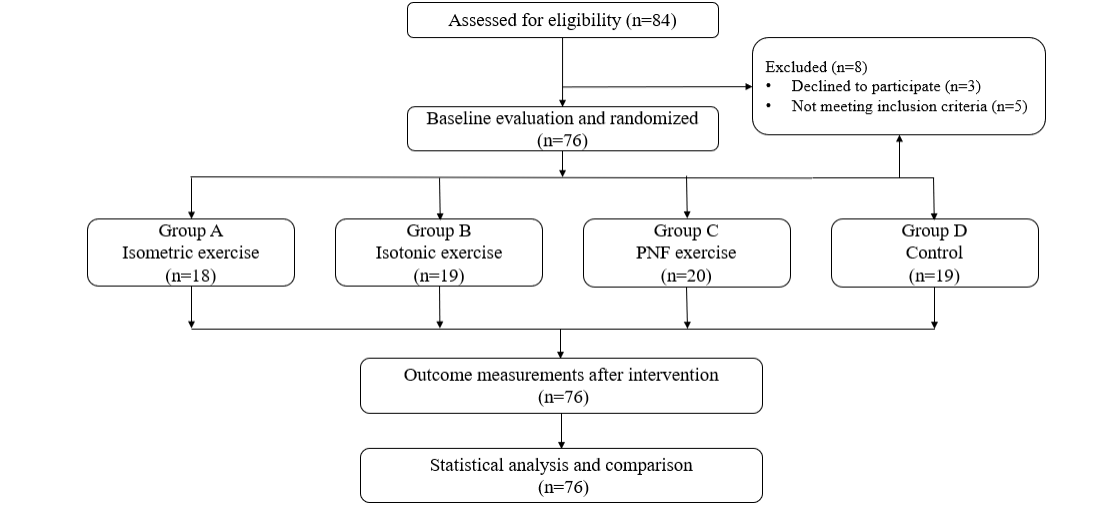
Flowchart of intervention. PNF: proprioceptive neuromuscular facilitation.

WZR and 2 other physical therapists evaluated all participants blinded to their exercise protocols. AN, XZH, and 1 other physical therapist guided all participants’ exercise interventions. All of the participants received compensatory exercise and manual therapies following the outcome measurement and statistical analysis (Figure 1).

### Interventions

#### Isometric Exercise

Participants in group A performed a modified isometric exercise program [34] including 2 scapula movements of scapula retraction (arm row at neutral position) and scapula elevation (dumbbell shrug at neutral position), and 1 shoulder abduction, where the dumbbells with adjustable weights were used. Scapula retraction and elevation were performed at 60% MVC or subpain threshold for 10 seconds with 15 sets at the neutral position. The shoulder isometric abduction was performed at 90°, and the elbow was flexed at 90°, with 60% MVC for 10 seconds of holding per 15 sets, and 30 seconds of rest was given between sets.

The MVCs of each movement were measured by a tension dynamometer with an LCD screen providing real-time statistics, where the participants were asked to perform a set of maximum voluntary contraction at the neutral position and adjust the contraction intensities by themselves during the exercise sessions. At 1 week before the intervention, this procedure was performed 3 times with a 30-second interval, and the mean value was identified as MVC.

#### Isotonic Exercise

Participants in group B performed a modified isotonic exercise program [35,36], including 2 scapula movements of dumbbell shrug and arm row and 1 shoulder lateral raise, where the dumbbells with adjustable weights were used. All of the isotonic exercises were performed at the moderate intensity [37] of 60% MVC or subpain threshold for 10 repetitions per 5 sets, where 1 minute of rest was given between sets.

#### PNF Exercise

Participants in group C performed a modified PNF exercise program [38,39], including the integration of agonist reversals (ARs), combination of isotonic contraction (CI), and rhythmic stabilization (RS) technique with the scapular pattern and upper extremity pattern. The upper extremity pattern D2 (flexion, abduction, and external rotation) was carried out using CI, where the starting position of the shoulder was 180° internal rotation, 45° adduction, and 0° extension, and the ending position of the shoulder was 0° external rotation, 135° abduction, and 180° flexion. During the CI training, participants were asked to perform the concentric shoulder external rotation, abduction, and flexion to the ending position, and perform the eccentric movements back to the starting position.

The scapular pattern D2 (anterior descending and posterior evaluation) was carried out using AR followed by RS. During the AR training, the starting position of the scapular was maximum protraction and downward rotation, and the ending position of the scapular was maximum retraction and upward rotation. Participants were asked to perform the concentric scapula retraction and upward rotation to the ending position, then finish the concentric reversal movements back to the starting position. Additionally, the scapular was set at a neutral position during the RS training, when the participants were asked to maintain the stability of the scapular and confronting the resistances from the physical therapist. All of the PNF exercise sessions performed 10 repetitions per 5 sets at approximately 60% MVC or subpain threshold, with 1 minute of rest between sets.

### Outcome Measures

#### PPTs of Trigger Points

PPTs of trigger points were measured by a quantitative sensory testing protocol [40] via a handheld pressure algometer (Baseline Dolorimeter, Fabrication Enterprises) with a 1 cm^2^ metal probe and applied at a rate of 0.5 kg/s. PPT was measured in the trigger point located in the upper trapezius, which was labeled by a sterile marker. Participants were instructed to report as soon as they perceived a pain intensity by the VAS score of 40 out of 100 (Pain40) during pressure application; then, that threshold was recorded as PPT. This test was performed 1 minute before and after the intervention, while the difference of PPTs during the exercise session was recorded as the local EIH responses.

#### PPTs of Remote Sites

PPTs of remote sites were measured at the point (5 cm below the lateral condyle of humerus) of extensor carpus radialis (test point of arms) and the point (10 cm below the lateral femoral condyle) of peroneus longus (test point of legs) ipsilateral to the exercise limbs and were performed 1 minute before and after each exercise session. The difference of PPTs during the exercise session was recorded as the remote EIH responses.

#### Conditioned Pain Modulation

The CPM response was measured by quantitative sensory testing protocol [41], with the test stimulation applied by pressure, and conditioned stimulation applied by cold water immerse [42]. Participants first received pressure stimulation at the ipsilateral extensor carpus radialis and report a PPT at Pain40 as a test stimulus. Then, participants were instructed to immerse the contralateral hand into cold water at 8 ℃ for 1 minute and report the PPT at Pain40 when the pressure applied again at the threshold after 30 seconds of immersing. The difference between the 2 PPTs was recorded as the response of CPM.

### Data Analysis

The main outcome of this study was the PPT of the trigger point, while the secondary outcomes were the PPT of the remote site and CPM responses. Normality of all data was assessed by means of the 1-sample Kolmogorov-Smirnov test. Difference in baseline data (height, weight, duration of pain, and VAS) between the groups was verified by the 1-way ANOVA test.

The 1-way ANCOVA was used to examine whether there was a significant difference within the 4 groups, considering PPT, EIH, and CPM at post exercise, while the pre-exercise measurements were set as covariates. The Bonferroni method was applied in the post hoc multiple comparison. The 2-tailed paired *t* test was used for the comparison within groups. All data were processed using SPSS, version 21.0 (IBM Corp), and the statistical significance was set at *P*<.05 for all tests.

## Results

### Baseline Characteristics

Of the 76 participants with MPS, 18 (24%) in group A completed the isometric exercise, 19 (25%) in group B completed the isotonic exercise, 20 (26%) in group C completed the PNF exercise, and 19 (25%) in group D finished a blank session. The average duration of shoulder MPS among participants was 7.56, 6.53, 6.85, and 7.53 weeks in group A, B, C, and D, respectively. The majority of participants (n=48, 63%) with MPS presented a duration greater than 6 weeks, and 33% (n=25) of them indicated over 8 weeks. Prior to the first exercise intervention, over half of the participants (n=44, 58%) had moderate-to-severe pain syndrome, with a VAS score higher than 40 mm/100mm. The baseline participant characteristics, including age (*P*=.95), height (*P*=.61), weight (*P*=.88), duration of pain (*P*=.54), and VAS (*P*=.18), did not present significant differences between the groups (Table 1).

**Table 1:** Baseline characteristics (N=76)^a^.

Measurements	A (n=18), mean (SD)	B (n=19), mean (SD)	C (n=20), mean (SD)	D (n=19), mean (SD)	*P* value
Age (years)	21.38 (2.50)	20.84 (1.92)	21.00 (1.81)	21.21 (2.41)	.95
Height (cm)	164.83 (3.75)	166.00 (4.53)	164.55 (4.21)	166.21 (4.44)	.61
Weight (kg)	55.03 (7.27)	57.32 (9.31)	54.75 (5.37)	59.57 (8.17)	.88
Duration of pain (week)	7.56 (2.83)	6.53 (2.95)	6.85 (2.52)	7.53 (3.01)	.54
VAS^b^ (mm)	45.08 (12.80)	43.76 (15.89)	43.67 (13.78)	42.63 (8.06)	.18

^a^1-way ANOVA; significant difference was set at *P*<.05.

^b^VAS: visual analog scale.

### The EIH and CPM Following Exercises

There was a significant increase in PPT at trigger point and arm site after isotonic (*P*<.001) and PNF exercises (*P*<.001), whereas the isometric exercise and the control group showed no difference compared to the baseline. The PPT at the arm sites significantly improved following PNF (*P*<.001) and isotonic exercises (*P*<.001), and the PPT at the leg sites also changed after isometric (*P*=.03), isotonic (*P*=.03), and PNF (*P*<.001) exercises.

**Figure 2:**
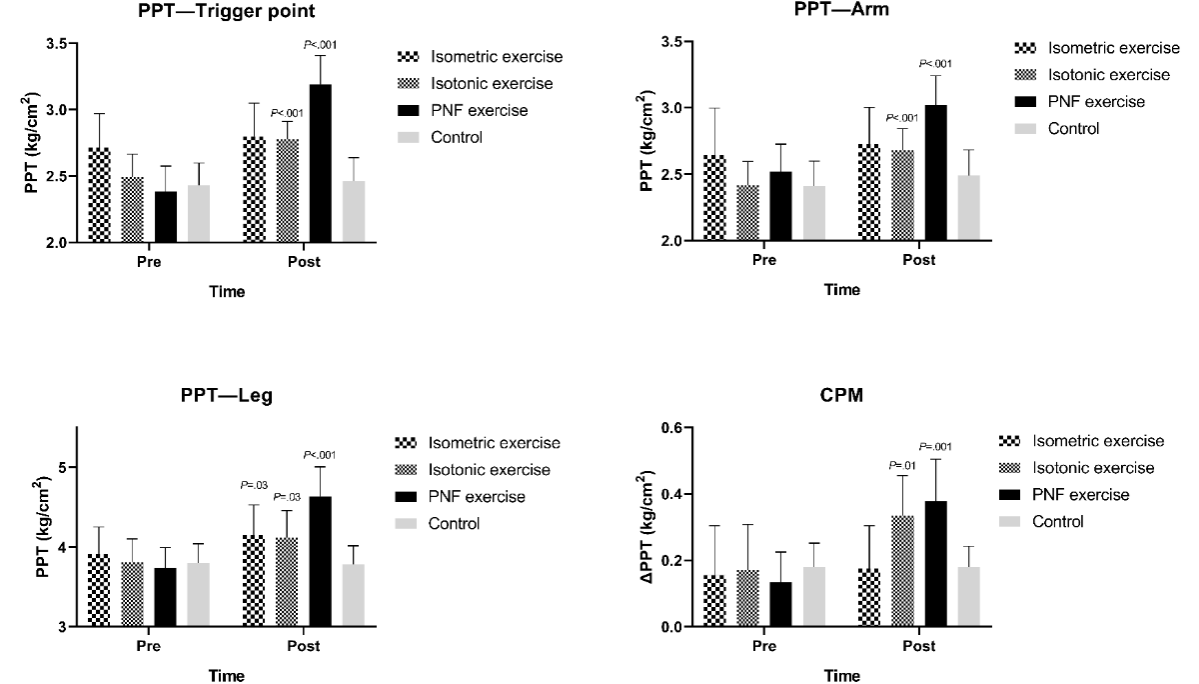
Results of pain pressure threshold (PPT), exercise-induced hypoalgesia, and conditioned pain modulation (CPM) following exercise interventions. PNF: proprioceptive neuromuscular facilitation.

A single session of isotonic (*P*=.01) and PNF (*P*=.001) exercises significantly improved the CPM responses, while the isometric exercise and the control group showed no difference compared to the baseline (Table 2 and Figure 2).

**Table 2:** Intervention results within groups (N=76)^a^.

Outcome measure and group			Before exercise, mean (SD)		After exercise, mean (SD)		*P* value
**PPT^b^—trigger point (kg/cm^2^)**							
	A	2.72 (0.51)		2.80 (0.50)		.41	
	B	2.49 (0.35)		2.78 (0.28)^c^		<.001	
	C	2.38 (0.41)		3.19 (0.47)^c^		<.001	
	D	2.43 (0.35)		2.46 (0.37)		.42	
**PPT—arm (kg/cm^2^)**							
	A	2.64 (0.71)		2.72 (0.56)^c^		.13	
	B	2.42 (0.37)		2.68 (0.34)^c^		<.001	
	C	2.52 (0.44)		3.02 (0.47)^c^		<.001	
	D	2.41 (0.39)		2.49 (0.40)		.15	
**PPT—leg (kg/cm^2^)**							
	A	3.91 (0.70)		4.15 (0.76)		.03	
	B	3.81 (0.61)		4.12 (0.70)^c^		.03	
	C	3.74 (0.55)		4.63 (0.80)^c^		<.001	
	D	3.80 (0.50)		3.78 (0.49)		.78	
**CPM^d^ (kg/cm^2^)**							
	A	0.16 (0.30)		0.18 (0.26)		.72	
	B	0.17 (0.28)		0.33 (0.25)^c^		.01	
	C	0.14 (0.19)		0.38 (0.27)^c^		.001	
	D	0.18 (0.15)		0.18 (0.13)		.91	

^a^Paired *t* test; significant difference was set at *P*<.05.

^b^PPT: pressure pain threshold.

^c^Significant changes.

^d^CPM: conditioned pain modulation.

### The Effect of Exercise Type on EIH and CPM

For the PPT of the trigger point site, both the PNF (*P*<.001) and isotonic exercises (*P*=.02) showed significantly higher increase compared to control group. Meanwhile, PNF exercise had a greater effect than isotonic (*P*<.001) and isometric (*P*<.001) exercises. However, there was no significant difference between the isotonic and isometric exercises (Table 3).

For the PPT of remote sites, only the PNF exercises increased significantly higher thresholds compared to the control group (*P*<.001 and *P*<.001), isotonic exercises (*P*=.01 and *P*=.004), and the isometric exercises (*P*<.001 and *P*=.002) at both arm and leg sites. For the CPM, only PNF exercise attained a significantly greater responses compared to both control group (*P*=.01) and isometric exercise (*P*=.02; Table 3).

**Table 3:** Between-group comparison results of the intervention^a^.

Outcome measures and intergroups				Mean difference		SE		*P* value		95% CI		
										Lower bound		Upper bound
**PPT^b^—trigger point (kg/cm^2^)**												
	**A**											
		B	–0.144		0.093		.76		–0.397		0.109	
		C	–0.638^c^		0.094		<.001		–0.894		–0.383	
		D	0.130		0.094		<.001		–0.126		0.385	
	**B**											
		C	–0.495^c^		0.090		<.001		–0.734		–0.243	
		D	0.274^c^		0.090		.02		0.028		0.519	
	**C**											
		D	0.768^c^		0.089		<.001		0.526		1.010	
**PPT—arm (kg/cm^2^)**												
	**A**											
		B	–0.123		0.086		.95		–0.351		–0.111	
		C	–0.389^c^		0.085		<.001		–0.618		–0.160	
		D	0.060		0.086		>.99		–0.174		0.294	
	**B**											
		C	–0.266^c^		0.083		.01		–0.492		–0.040	
		D	0.183		0.084		.20		–0.045		0.411	
	**C**											
		D	0.449^c^		0.083		<.001		0.223		0.675	
**PPT—leg (kg/cm^2^)**												
	**A**											
		B	–0.053		0.165		>.99		–0.500		0.395	
		C	–0.623^c^		0.163		.002		–1.067		–0.180	
		D	0.285		0.165		.53		–0.162		0.733	
	**B**											
		C	–0.570^c^		0.160		.004		–1.006		–0.135	
		D	0.338		0.162		.25		–0.103		0.779	
	**C**											
		D	0.908^c^		0.160		<.001		0.473		1.344	
**CPM^d^ (kg/cm^2^)**												
	**A**											
		B	–0.152		0.069		.19		–0.339		0.034	
		C	–0.212^c^		0.068		.02		–0.397		–0.027	
		D	0.007		0.069		>.99		–0.181		0.194	
	**B**											
		C	–0.061		0.067		>.99		–0.243		0.122	
		D	0.159		0.068		.14		–0.026		0.344	
	**C**											
		D	0.219^c^		0.067		.01		0.036		0.402	

^a^1-way ANCOVA, adjusted by Bonferroni. Significant difference was set at *P*<.05.

^b^PPT: pressure pain threshold.

^c^Significant changes.

^d^CPM: conditioned pain modulation.

## Discussion

### Principal Findings

This pilot study investigated the local and remote responses of EIH and CPM after PNF, isotonic, and isometric resistance exercises for patients with MPS. Our findings mostly met what we previously hypothesized. PPT was increased at trigger point, arm, and leg sites when participants performed PNF and isotonic exercise, but was only increased at leg sites when participants performed isometric exercise. Compared with the control group, both isotonic and PNF groups showed significant greater EIH responses at the trigger points. However, only the PNF exercise significantly improved PPT at remote sites and CPM responds compared to the control group.

### Changes in Pain Modulation

MPS, generally regarded as a typical chronic musculoskeletal pain [43], is mainly characterized by the presence of trigger points [44], which is a hypersensitive area that can be palpated in a muscle taut band. The trigger point is possibly induced by a continuous nociceptive stimulus from the local energy crisis of overused muscle fibers [45], followed by pain sensitization [46], which means the impairment of descending pain modulation. Thus, chronic MPS could attenuate both the CPM and the EIH effect. Vaegter et al [47] reported an altered effect of EIH in patients with chronic low back pain, where the acute 6-minute walk failed to induce EIH in patients with greater pain sensitivity. Chretien et al [5] also found that the deficits of EIH were related to the reduced CPM among adolescent girls with chronic pain.

This study found that both PNF and isotonic exercises significantly improved the CPM responses of MPS patients. It suggested that exercise with optimal intensity and type could affect central pain modulation and mediate neurotransmitters and cytokines. Exercises with various resistances may enhance the descending inhibition and reduce pain by activating the endocannabinoids, endogenous opioids, and 5-HT system. Crombie et al [48] reported that the serum endocannabinoids increased alongside the attenuation of pain sensitivity after resistance exercises in healthy individuals, while Bobinski et al [13] also found an increase in 5-HT in rostral ventromedial medulla through low-intensity exercise. Meanwhile, the activation of pain-related cortex regions following exercises may also mediate the pain processing of the thalamus and periaqueductal gray. Cummiford et al [49] found that the pain perception and facilitation of the thalamus was constrained by primary motor cortex stimulation, while Ellingson et al [50] reported that the DLPFC function improved and was correlated with pain reduction in patients with fibromyalgia after cycling exercise. Interestingly, Lial et al [51] found that PNF exercise significantly improved DLPFC activation, which may indicate potential impacts on the central pain modulation, and that it still needs further investigation.

### Changes in Pain Perception

In this study, EIH was demonstrated at the trigger point, arm, and leg sites for patients with MPS following PNF and isotonic exercises, which is consistent with prior research under various painful conditions. In most cases, resistance exercises with optimal intensities can induce global and local analgesia effects. First, the global analgesic effects induced by PNF and resistance exercises were also verified by Burrows et al [23]; these findings showed that the isotonic shoulder exercises of nonpainful limbs in patients with knee osteoarthritis effectively reduced pain, while Kuppens et al [52] confirmed EIH responses at leg sites after moderate-intensity shoulder extension exercises. Koltyn er al [53] also demonstrated the EIH responses elicited by contralateral isometric contraction in healthy individuals. Second, the isotonic and PNF exercises elicited greater EIH than isometric exercises, which is also found by Chung et al [54], showing that the isotonic exercises have superior EIH responses than isometric exercises in patients with chronic neck pain. Lastly, only isometric exercise failed to attenuate the pain perception of trigger point, which is also consistent with a previous systematic review by Bonello et al [55], indicating that there is no consistent evidence for EIH following isometric exercises in patients with chronic pain. Staud et al [56] also found that isometric exercise increased pain intensity in patients with fibromyalgia.

Neuromuscular exercises such as PNF have a significant therapeutic effect on many musculoskeletal pain conditions. According to a meta-analysis by Gao et al [18], PNF has more beneficial effects on pain relief and waist function improvement in patients with chronic lower back pain than other exercise interventions. Regarding chronic neck pain, Lytras et al [57] found that neuromuscular inhibition therapy combined with exercise intervention effectively reduced pain rating and improved neck function, while PNF also had a significant effect on knee osteoarthritis [58] and patellofemoral pain syndrome [59].

### Potential Mechanisms of EIH

In this study, PNF exercise had a greater analgesic effect on MPS after intervention compared to control group and other exercises. This may be explained by the enhanced proprioception and C fiber inputs from the additional eccentric [38] and dynamic muscle contractions. Although the activation of noxious C fibers following the overload exercise [19] may trigger the mechanical allodynia [60] and delayed onset muscle soreness, the sufficient nonnoxious C fiber input during the eccentric contraction with subpain threshold intensity in the PNF or even the isotonic exercise may still activate the descending inhibition via thalamus ventromedial nucleus. Stackhouse et al [61] compared the analgesic effect between the noxious electrical stimulation and the eccentric plantar flexor exercise with moderate intensity, and found that eccentric exercise induced both mechanical and thermal pain perception effectively. Apart from the enhanced proprioception and the C fiber inputs during the PNF exercise, the interaction between the participants and the researcher may also have positive effects on the proprioception inputs, considering the resistance provided by manual contact and the personal adaptation from the physical therapist. In such conditions, the intensity and direction of resistance can be adjusted more relevant to patients’ perception.

Apart from the PNF exercise, only the isotonic exercise showed a greater change of PPT compared to control group at the trigger point, implying that the onset hypoalgesia effect from the CPM test may have contributed these changes during the test. The CPM test applied in this study, which provided cold stimulus, can also activate the C fiber afferent and the descending inhibition [41], which may have the overlap effect with the EIH responses. Thus, the relationship between the CPM test and the exercise requires further investigation.

### Limitations

This study has several limitations. First, the indicators of the pain tests were limited; for instance, the PPT combined with thermal pain thresholds might better reflect the real neurophysiological aspects of musculoskeletal pain. Second, individuals with MPS differed in terms of pain duration and intensity, which may have an impact on the consistency of the results. Third, all of the participants were young female students, so the possible gender and aging difference of the pain processing should be considered in future studies. Lastly, the EIH effectiveness of moderate-intensity resistance exercise in this work was insufficiently investigated, and still needs to be evaluated comprehensively by increasing the variety of exercise types and duration length of the interventions.

### Conclusions

In summary, PNF, isotonic, and isometric exercises could exert significant local and global EIH effects for patients with MPS, which may be influenced by the proprioception stimulus under the exercise types. The significant increases in CPM response after PNF and isotonic exercises indicated that the EIH mechanisms of these moderate-intensity exercises may involve the enhancement of the central descending inhibitory function. The findings of this study can serve as theoretical foundations for further studies focusing on central mechanisms of EIH, which could optimize the effect of exercise interventions for chronic pain in future clinical practices.

## Appendix

Multimedia Appendix 1 CONSORT-eHEALTH checklist (V 1.6.1).

## Abbreviations

5-HT: 5-hydroxytryptamine
AR: agonist reversal
CI: combination of isotonic contraction
CPM: conditioned pain modulation
EIH: exercise-induced hypoalgesia
MVC: maximum voluntary contraction
PNF: proprioceptive neuromuscular facilitation
PPT: pressure pain threshold
RS: rhythmic stabilization
VAS: visual analog scale
